# Do the COVID-19 Crisis, Ageing and Climate Change Put Swiss Fiscal Sustainability at Risk?

**DOI:** 10.1007/s10272-022-1027-8

**Published:** 2022-02-12

**Authors:** Thomas Brändle, Pierre-Alain Bruchez, Carsten Colombier, Martin Baur, Lukas Hohl

**Affiliations:** Eidgenössische Finanzverwaltung EFV, Bundesgasse 3, 3003 Bern, Switzerland

**Keywords:** H60, J11, Q54

## Abstract

The ongoing coronavirus pandemic crisis as well as demographic and climate change pose major challenges for public finances. This article deals with the implications of demographic trends in Switzerland, i.e. the progressive ageing of the population and its impact on the country’s public finances in the long run. As the analysis shows, the brunt of the demographic burden is borne by the old-age pension scheme, health and long-term care. This article also addresses the financial ramifications of the COVID-19 crisis and shows the need for economic policy action over the longer term to ensure the sustainability of public finances in Switzerland. Furthermore, a qualitative assessment of climate change is included, as it constitutes an additional major long-term challenge for public finances.

## Methodological principles

In accordance with the international standard set by the European Commission, the Organisation for Economic Co-operation and Development and the International Monetary Fund (IMF), we apply a cohort simulation approach to analyse how demographic change will affect the sustainability of Switzerland's public finances (European Commission, 2021; IMF, 2021).[1] As its goal is to assess the need for action, this analysis is based on the hypothesis that the legal status quo and the reforms already set out in the 2023-2025 Confederation's financial plan will last from 2019 to 2050 ("no policy change").

To evaluate the long-term fiscal impact of population ageing, we project the age-dependent public expenditure on ageing, disability, education, health and long-term care. The reference population scenario for the period 2020-2050 of the Federal Statistical Office (FSO) provides the foundation for these projections (FSO, 2020). Apart from the COVID-19 crisis and interest expenditure, non-demographic public expenditures and public revenues are generally assumed to increase proportionally to GDP. However, the current annual profit distribution by the Swiss National Bank (SNB) to the Confederation and the cantons is fixed at CHF 6 billion until 2050.

As this analysis is based on projections of long-term development, it inevitably involves uncertainties. Therefore, the results are to be understood as "if-then" hypotheses: if demographics and the economy develop as assumed, then this will mean an additional burden for public budgets due to increased expenditure on health care, for example. Neither repercussions of public finances on macroeconomic behaviour nor the implications of the expected slow-down in growth of the labour force on productivity and wages are taken into account.

## Macroeconomic assumptions

Due to the high degree of uncertainty about long-term effects of the COVID-19 crisis, two different economic scenarios are projected for economic development up to

1 For a comprehensive treatise see the latest sustainability report by the Federal Department of Finance (2021).

**Thomas Brändle**, Federal Finance Administration, Bern, Switzerland.

**Pierre-Alain Bruchez**, Federal Finance Administration, Bern, Switzerland.

**Carsten Colombier**, Federal Finance Administration, Bern, Switzerland.

**Martin Baur**, Federal Finance Administration, Bern, Switzerland.

**Lukas Hohl**, Federal Finance Administration, Bern, Switzerland.

Figure 1
**Real GDP for 2017-2020 and in the positive and negative scenarios**
in CHF million

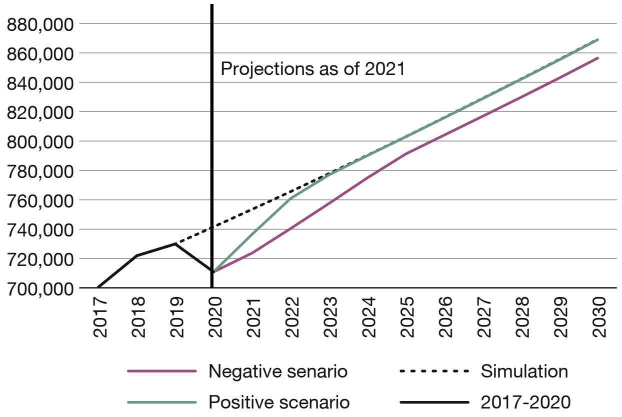

Sources: State Secretariat for Economic Affairs (SECO); Federal Finance Administration.

Figure 2
**Old-age dependency ratios**

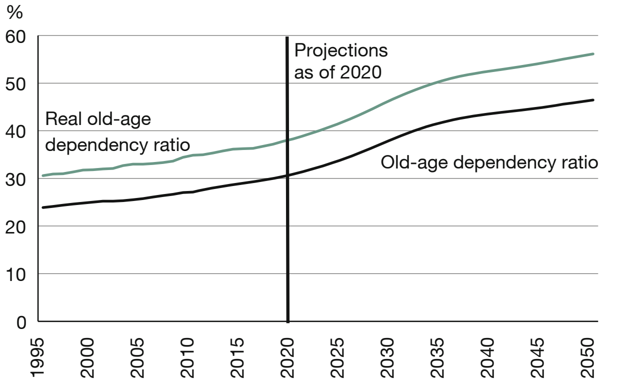

Note: Old-age dependency ratio: number of people over 65 relative to the working-age population. Real old-age dependency ratio: number of people over 65 relative to the labour force, full-time equivalents.

Source: Federal Statistical Office.

2025: a positive scenario, which assumes a rapid return to the pre-crisis level of the baseline year 2019, and a negative scenario, in which a sluggish recovery is assumed (see Figure 1). Because of the slow recovery, the simulated pre-crisis level of real GDP is not reached over the entire projection period in the negative scenario. While the average annual growth rate of real GDP amounts to 1.6% between 2020 and 2025 in the positive scenario, the respective growth rate is equal to 1.4% in the negative scenario.

In the long run (from 2026), real GDP grows on average by 1.5% annually in both scenarios. Based on past developments, we assume that the annual productivity growth is equal to 1.2% and the equilibrium long-term real interest rate is set at 1.6%. To reflect the current low-interest rate environment, it is additionally assumed that the equilibrium long-term interest rate will not be reached before the beginning of the 2030s. We presuppose that the yearly inflation rate equals 1.0% and wages increase proportionally to productivity growth.

## Demographic development

According to the FSO's reference population scenario, the population should increase from 8.7 million in 2020 to just under 10.4 million in 2050, which corresponds to a mean annual growth rate of 0.6% (FSO, 2020). However, population growth slows down during the projection horizon. Life expectancy at birth rises from 83.7 years in the baseline year to 88.4 in 2050. While the share of the working-age population (people aged 20-64) shrinks from 61% to 55%, the share of people aged 80 and above more than doubles from 5% to 11% over the projection horizon.

An important factor in determining population and labour force growth – but one that is difficult to estimate – is future net migration (the number of immigrants less the number of emigrants each year). The FSO reference scenario assumes net migration of 50,500 people in 2020. Net migration increases to 55,000 people until 2029, before falling to 35,000 people by 2040 and then remaining constant until the end of the projection period.

The old-age dependency ratio shows the number of people over 65 relative to the working-age population (see Figure 2). Whereas in 1995 there were just over four persons of working age for each person over the age of 65 (old-age dependency ratio 23.9%), by 2020 this number had fallen to 3.2 persons (old-age dependency ratio 30.9%). This ratio declines to 2.1 persons (old-age dependency ratio 46.5%) in 2050. The real or "effective" old-age dependency ratio is the number of pensioners relative to the number of people in the labour force, measured in full-time equivalents. This ratio is thus higher than the old-age dependency ratio. The retirement of the baby boomers between 2020 and 2035 causes the strongest increase in old-age dependency ratios as society will age fastest during this period.

Figure 3
**General government expenditure in the past and in the positive scenario**
in % of GDP

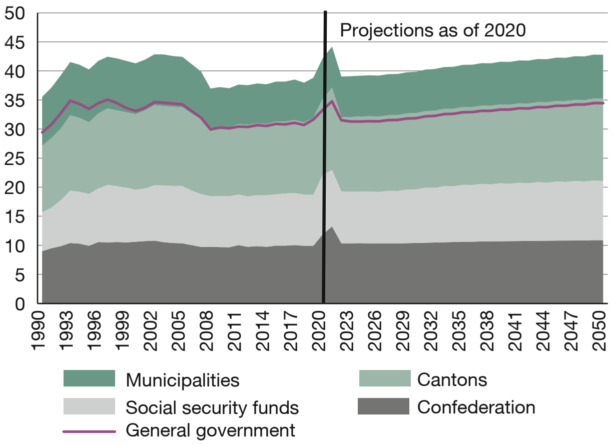

Source: Federal Finance Administration.

Figure 4
**Age-dependent expenditure by function in the positive scenario**
in % of GDP

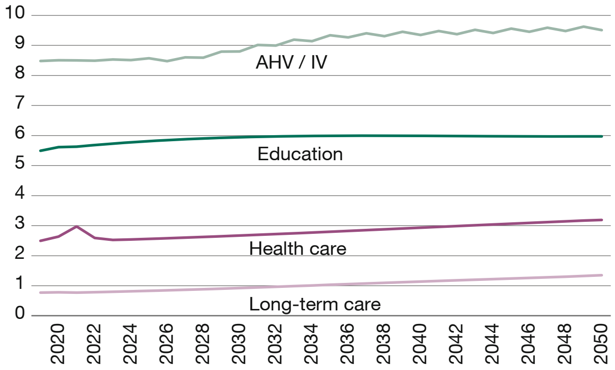

Notes: AHV stands for social old-age and survivors' pension insurance; IV stands for social disability insurance; differences are due to rounding. The zigzag shape of the AHV/IV expenditure curve stems from the biannual indexation of pensions (mixed index). The mixed index equals the weighted average of real-wage growth (weight = 0.5) and inflation (weight = 1).

Source: Federal Finance Administration.

## COVID-19 crisis, ageing and general government expenditure

According to the projections, the COVID-19 crisis caused general government expenditure to rise temporarily from 31.6% to 34.8% of GDP in 2020 and 2021 (see Figure 3).[2] Thereafter, in the positive scenario, the general government expenditure ratio returns to virtually the same level as in 2019. More than 80% of the pandemic-related additional expenditure (e.g. short-time working, hardship assistance, coronavirus tests and vaccinations) is borne by the Confederation. The remainder is largely borne by the cantons.

However, the general government expenditure ratio increases in the long run due to demographic change. Between 2019 and 2050, it climbs from 31.6% to 34.5% of GDP in the positive scenario. This is almost entirely due to age-dependent expenditure such as old-age and survivors' insurance, disability insurance, health care, long-term care and education.

The lower level of GDP in the negative scenario is barely felt in the long run. General government expenditure rises only 0.5% of GDP faster than in the reference scenario and has a similar shape.

2 In the baseline year of 2019, 1% of GDP corresponds to CHF 7 billion.

## Old-age pension scheme and cantons most severely affected by ageing

The most important impact of ageing on public expenditure in the positive scenario can be seen in the area of old-age and disability provision (AHV and IV), almost exclusively driven by the retiring baby boomers until 2035 (1.0% of GDP), followed by health and long-term care (0.7% and 0.6% of GDP respectively), as shown in Figure 4. Government-financed outlays for COVID-19 cause a spike in health care expenditure of 0.5% of GDP between 2019 and 2021. Education expenditure as a percentage of GDP steeply increases by a fast-rising number of students until 2035 (0.5% of GDP). Afterwards, this increase flattens out so that education expenditure as a percentage of GDP remains constant until 2050.

The responsibilities of the levels of government for the various age-dependent expenditure areas determine the extent to which the individual levels of government are impacted by the ageing of society (see Table 1).

According to the projections, the Confederation (through old-age provisions), social security funds and cantons bear the brunt of demographic change. The cost driver for the Confederation and social security funds is AHV expenditure triggered by ageing. With the retirement of the baby boom generation up to the mid-2030s, the biggest increase continues until 2035, de-

Table 1
**Age-dependent expenditure by level of government in the positive and negative scenarios**
in % of GDP

| | 2019 | 2035 | | | 2050 | | |
|---|---|---|---|---|---|---|---|
| Government level | Baseline year | Positive | Negative | Difference | Positive | Negative | Difference |
| Confederation | 3.8 | 4.4 | 4.5 | 0.10 | 4.5 | 4.6 | 0.10 |
| Social security funds | 5.5 | 5.9 | 5.9 | 0.05 | 6.1 | 6.1 | 0.05 |
| AHV | 4.7 | 5.2 | 5.3 | 0.04 | 5.5 | 5.5 | 0.04 |
| IV | 0.8 | 0.6 | 0.6 | 0.01 | 0.5 | 0.5 | 0.00 |
| Cantons | 5.5 | 6.2 | 6.2 | 0.08 | 6.6 | 6.7 | 0.08 |
| Municipalities | 2.4 | 2.7 | 2.7 | 0.05 | 2.8 | 2.8 | 0.05 |
| General government (consolidated) | 17.2 | 19.3 | 19.5 | 0.26 | 20.1 | 20.4 | 0.26 |

Notes: AHV stands for social old-age and survivors' pension insurance; IV stands for social disability insurance; differences are due to rounding.

Source: Federal Finance Administration.

spite the AHV 21[3] reform. According to the projections, this rise amounts to 0.6% of GDP for the Confederation and 0.4% of GDP for the social security funds. An additional factor at the federal level is the increase in AHV transfer expenditure due to the TRAF (Tax reform and AHV financing). Relative to GDP, expenditure rises by a moderate 0.1% for the Confederation and 0.2% for the social security funds between 2035 and 2050. The positive development of disability insurance expenditure attenuates the social security funds' expenditure increase.

Age-dependent cantonal expenditure is determined by health care, long-term care and education. Up to 2035, the demographic pressure comes mainly from health care and education expenditure (0.7% of GDP). After 2035, health care and long-term care are the key expenditure drivers (0.4% of GDP). The communes are less affected. Their expenditure increases from its current level of 2.4% of GDP to 2.8% by 2050, driven by education and long-term care. Due to the declining growth in pupil and student numbers, education expenditure no longer puts pressure on the cantonal and communal finances from 2035 onwards.

In the negative scenario, the same demographic burden leads to a slightly higher expenditure increase of 0.3% of GDP, as this scenario factors in a lower level of GDP. This additional burden is distributed evenly among the Confederation, cantons and communes. The social security funds' expenditure relative to GDP remains virtually unchanged, as the more pessimistic economic forecast from 2021 to 2025 curbs the increase in AHV/IV expenditure as well as economic growth.

## Ageing puts fiscal sustainability at risk

In line with the international standard, we consider fiscal policy as sustainable, if the public debt-to-GDP ratio can be stabilised at a specified target level, usually the debt ratio of the baseline year, in our case 2019 (Blanchard, 1990; IMF, 2021).[4]

The increase in age-dependent expenditure means that current benefit entitlements will have to be lowered in the future and/or financed by higher taxes and/or social security contributions. If steps are not taken to restore the financial equilibrium between receipts and expenditure, government indebtedness will rise from 25.4% to 45.0% of GDP between 2019 and 2050 (see Figure 5).

The debt ratio initially rises to 31.7% of GDP until 2021 because of the pandemic. It then falls to a low of 23.3% of GDP by 2032. This decrease is due to the assumed rapid economic recovery following the COVID-19 crisis. Moreover, the AHV runs at a surplus until 2028 thanks to the TRAF and AHV 21 reforms, in spite of the added cost pressure brought by the retirement of the baby boom generation. In addition, the debt servicing of the public purse is assumed to remain low until the end of

3 The AHV 21 is a reform proposed by the federal government that is currently debated in the Swiss parliament. Main elements are the alignment of women's retiring age with that of men (from 64 to 65 years) and an increase of the value-added tax by 0.7 percentage points in favour of AHV.

4 The federal debt-break rule imposes a stricter definition of fiscal sustainability (Federal Department of Finance, 2021). The Confederation's nominal debt level has to be stabilised.

Figure 5

**Debt ratio by government level in the positive and negative scenarios**

in % of GDP

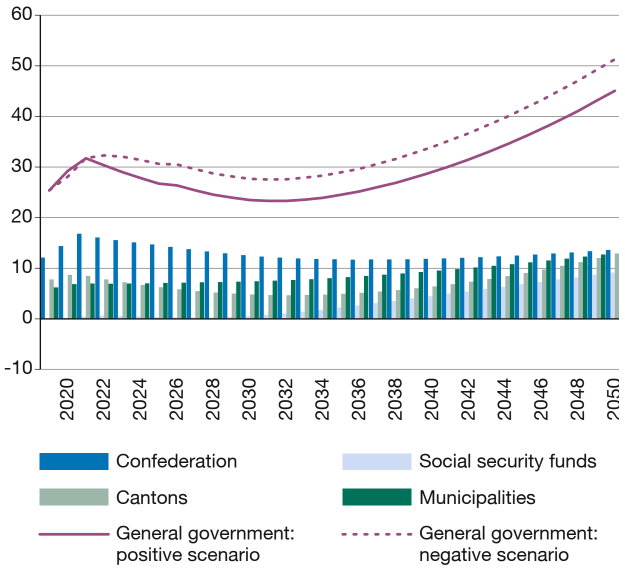

Source: Federal Finance Administration.

the 2020s due to the low interest rate environment. The government debt ratio increases from 2033, primarily because of demographic factors combined with a lessening impact of the AHV 21 reform, and reaches 45% of GDP by 2050.

The assumed profit distribution (over and above the basic distribution amount) by the SNB to the Confederation and cantons benefits public finances and the debt ratio over the entire projection period.

The debt ratio initially increases more in the negative scenario because of weaker economic growth during the COVID-19 crisis. In 2021, the debt ratio rises to 32.9% of GDP. The government debt ratio then also recovers subsequently – albeit not as much as in the positive scenario. The interim lowest point is reached in 2031 at 27.5% of GDP. The debt ratio then rises to 51.2% of GDP by 2050.

Public finances are not sustainable due to the rising debt ratio, resulting in a fiscal gap for the general government. This measure indicates the degree to which permanent savings, contribution increases or tax hikes will be necessary from 2025 onwards in order for the debt ratio to be stabilised at the level of the baseline year of 2019 by 2050. As shown in Table 2, in the positive scenario, the general government's fiscal gap is 0.8% of GDP, versus

Table 2

**Fiscal gap for the stabilisation of the public debt-to-GDP ratio in the positive and negative scenarios**

% of GDP

| | 2025-2050 annual consolidation needs | |
|---|---|---|
| Government level | Positive | Negative |
| Confederation | 0.1 | 0.4 |
| Social security funds | 0.4 | 0.5 |
| Cantons | 0.2 | 0.4 |
| Municipalities | 0.3 | 0.3 |
| General government | 0.8 | 1.1 |

Source: Federal Finance Administration.

1.1% of GDP in the negative scenario.[5] It is also noteworthy that the fiscal gap increases if the start of budget consolidation is postponed until after 2025. The longer the budget consolidation is postponed, the greater the need to bring public finances back into line.

The sustainability of the AHV is most at risk. It has the highest fiscal gap at 0.5% of GDP. The AHV 21 reform can keep the AHV balanced up to 2028. However, the AHV moves into deficit thereafter. The positive development of disability insurance narrows the fiscal gap, totalling 0.4% of GDP for the social security funds. In the negative scenario, the fiscal gap rises only moderately to 0.5% of GDP.

Notwithstanding the added burden of the COVID-19 crisis, the Confederation can just about maintain a sustainable fiscal policy in the positive scenario, with a fiscal gap amounting to 0.1% of GDP. Without the supplementary distributions from the Swiss National Bank (SNB) to the Confederation of CHF 1.3 billion per annum, the fiscal gap would double to 0.2% of GDP. In the negative scenario, the fiscal gap rises to 0.4% of GDP, as the COVID-19 crisis leads to significantly lower receipts assuming the same demographic burden.

The cantons have an annual fiscal gap of 0.2% of GDP until 2050 caused by the expenditure pressure for health care and long-term care. This is relatively low, not least because of the assumed annual profit distribution by the SNB to the cantons totalling CHF 4 billion. Without the SNB's supplementary distributions of CHF 2.7 billion per annum, the fiscal gap would double to 0.4% of GDP; this also applies in the negative scenario. That is due to the assumption that, unlike in the positive scenario, receipts

5 For a debt-brake consistent fiscal gap see Federal Department of Finance (2021).

Figure 6
**Disposable income per capita, 1990-2050, in the positive and negative scenarios (at 2019 prices)**
in CHF

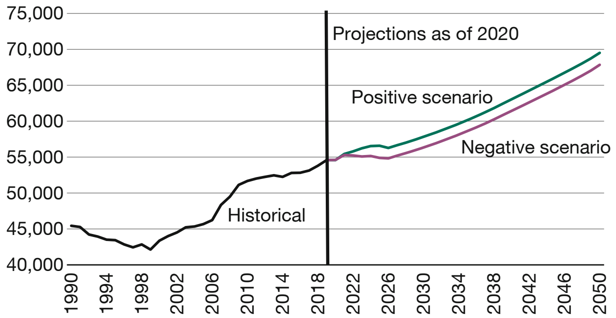

Sources: Federal Finance Administration; Federal Office of Public Health; State Secretariat for Economic Affairs (SECO).

will be lower. The situation is similar for the communes: their fiscal gap amounts to 0.3% of GDP in the positive scenario. In the negative scenario, it is just under 0.1% of GDP higher than in the positive scenario.

## Productivity growth makes future generations richer

The disposable income per capita of the Swiss population is used to show how strong the additional burden is felt on average by Swiss residents if the debt ratio of the general government is stabilised at its current level, with the additional age-dependent expenditure financed through higher taxes and social security contributions. The disposable per capita income is displayed as the inflation-adjusted average income per capita after the deduction of taxes, social security contributions and compulsory health insurance premiums (see Figure 6).

Despite the additional expenditure needed to close the fiscal gap, per capita disposable income continues to rise on average, driven by annual productivity advances of 1.2%. In the positive scenario, the projected annual rise between 2019 and 2050 (0.8%) is slightly higher than for the period 1990-2019 (0.7%), as growth was weak in the 1990s. The projected annual increase is 0.7% between 2019 and 2050 in the negative scenario.

Overall, it can be concluded that the Swiss population will experience a significant increase in its prosperity despite the growing financial burden of the ageing population. That holds true for both the positive and negative scenarios.

Table 3
**Budget items affected by climate change**

| Receipts | Public expenditure |
|---|---|
| • Greenhouse gas levies ($CO_2$ tax)<br>• Mineral oil taxes<br>• Revenue from enterprises affiliated with the Confederation<br>• Other taxes (VAT, income taxes, etc.) | • Infrastructure protection<br>• Energy efficiency of public buildings<br>• Subsidies to mitigate climate change<br>• Subsidies to adapt to climate change and repair the damage caused<br>• Research and development<br>• Negative emissions financing<br>• Foreign aid in the area of climate change |

Source: Federal Finance Administration.

## Climate change as a further challenge for public finances

Relative to the effects of demographic change, it is difficult to quantify the effects of climate change on public finances, as there are still numerous variables. The physical consequences of climate change are uncertain given the complexity of the earth system, specifically the feedback effects that can diminish or amplify the initial impacts. These uncertainties comprise extreme risks, such as tipping points and domino effects. As regards the economic consequences, it is practically impossible to predict technological innovation and economic structure over the long run. It is particularly difficult to assess whether and to what extent the economy will be able to adapt to the new conditions. In the absence of an orderly transition, impacts on the economy could be magnified by financial crises and political conflicts. Vöhringer et al. (2017) deliver the most up-to-date estimation of the impact on the Swiss economy.[6] According to the authors, however, their study is still incomplete. Given the incompleteness of the quantification of the economic impact in Switzerland, it is not yet possible to quantify the fiscal impact.[7]

6 There are other quantitative estimations of the impact of climate change on the Swiss economy. For example, Swiss Re (2021) evaluates the impact of the physical risks emanating from gradual climate change over time on GDP, and vulnerability to extreme weather risks (wet and dry conditions). They estimate that out of 48 major economies, Switzerland will be the second least affected (after Finland) by climate change by the middle of the century.

7 The UK Office for Budget Responsibility (2021) has attempted to quantify the consequences of climate change for the United Kingdom's public finances. Quantitative evaluations were also made previously, for example for the public finances of Germany (Infras and Ecologic, 2009) and Austria (Bachner and Bednar-Friedl, 2019). See also Baur et al. (2021) for further discussion.

Because of these uncertainties, our assessment of the fiscal impact remains qualitative. Climate change is likely to have a mostly negative impact (see Table 3). It may reduce the scope for fiscal policy leeway through lower tax receipts, additional expenditure and higher financing costs for government debt. The fiscal impact depends on the extent to which the costs of climate change are borne by the private sector. It also depends on the measures implemented to mitigate climate change: Regulation does not have the same impact on the budget as subsidies or $CO_2$ taxes. It is also to be expected that liability risks resulting from extreme situations will become more of an issue for public finances.

It is important to distinguish between the effects of climate change itself and the effects associated with mitigation and adaptation measures. The latter will initially probably cost more than the impact of climate change itself. The revenue of the tax on mineral motor oil will fall, in particular because of the rising market share of electric vehicles. More broadly, mitigation measures will have economic consequences and therefore will have an effect on public finances, e.g. on GDP-dependent tax receipts such as value-added tax or income tax. Insofar as climate change also brings about innovations in the area of green technologies, growth in those sectors could partly offset the decline in tax receipts. The state will also have to contribute directly to mitigation, for example as the owner of public buildings in need of energy renovation. A $CO_2$ tax is key to mitigating climate change. It is, however, a regressive tax as energy costs constitute a larger part of the budget for low-income households. This is why most of the $CO_2$ tax revenue in Switzerland is redistributed to households and firms. To the extent that the revenue is redistributed, it has no direct budgetary consequences, as it does not interact with other items of the budget. However, part of the $CO_2$ tax revenue is used to finance subsidies for mitigation. Mitigation or removing greenhouse gases from the atmosphere will eventually have to be financed directly by the public budget as $CO_2$ tax revenue diminishes in tandem with the fall of fossil fuel energy consumption.

The adjustment to the gradual increase in the average temperature in Switzerland should be manageable on the whole through to 2050. According to a study conducted by the Swiss Federal Institute of Technology (ETH), in 2050 Zurich will have the same climate as Milan does now (Bastin et al., 2019). This suggests that the consequences attributable solely to the incremental temperature increase in Switzerland will probably be relatively mild up to 2050. However, the warming will not stop in 2050 and the effects will be more pronounced in rural areas outside the cities where the activities (agriculture, tourism, etc.) are more dependent on the natural environment.

It is harder to estimate the effects of extreme weather events. Firstly, because the extent of the connection between climate change and certain extreme weather events, although proven, still needs to be researched in more detail.[8] Secondly, because adaptation measures required for relatively rare events are less likely to be implemented compared to adaptation to an incremental increase in the average temperature. In Switzerland, extreme weather events can cause flooding, landslides, avalanches, hail or, by contrast, heatwaves and drought. The consequences of the extreme weather events may be greater than those of the incremental average temperature increase; however, Switzerland will likely be exposed to events to which it is largely able to adapt.

As other countries will be more affected by climate change, the consequences of the effects felt by the rest of the world are likely to prove to be a significant channel of impact on Switzerland over the longer term. The effects on international trade are likely to be keenly felt in Switzerland, a small and open market economy. However, Switzerland's main trading partners, such as Germany, are not among those most affected by climate change. Another tangible effect of climate change could be an increase in foreign aid and immigration from severely impacted parts of the world.

The points raised above refer to likely scenarios. Given the high level of uncertainty, however, less likely scenarios must also be considered. There are threshold values or limits, known as tipping points, the crossing of which would unleash a vicious circle with ever more devastating consequences. One example would be the release of methane (a greenhouse gas) from the thawing of the Arctic permafrost. The possibility of such extreme scenarios is even more disturbing given that some consequences may be irreversible. Taking account of these extreme scenarios should lead to more forceful measures to mitigate climate change. The state, however, should avoid focusing solely on these risks at the expense of other important issues.

The first consideration in addressing the risk of climate change on public finances is to curb greenhouse gas

8 According to the IPCC (2021), "Many changes in the climate system become larger in direct relation to increasing global warming. They include increases in the frequency and intensity of hot extremes, marine heatwaves, and heavy precipitation, agricultural and ecological droughts in some regions, and proportion of intense tropical cyclones, as well as reductions in Arctic sea ice, snow cover and permafrost".

emissions in Switzerland by making the most efficient use of economic policy instruments such as incentive taxes, subsidies and regulations. From a fiscal policy perspective, it is important to maintain low public debt and balanced public finances, as they both allow room for manoeuvre for fiscal policy in order to be able to deal appropriately with the uncertain effects of climate change.

## Need for economic policy action

This article shows that public finances will be put under significant strain by the ageing population over the next three decades. The impact of ageing will be particularly noticeable through to 2035 as baby boomers retire. Even if the AHV 21 reform is implemented as planned, new reforms will be needed no later than the beginning of the 2030s to ensure a sustainable AHV. Due to the persistently high financial pressure in the health care and long-term care sectors, the age-dependent expenditure of the cantons will increase the most. The assumed profit distribution by the SNB (CHF 6 billion, with 2 billion for the Confederation and 4 billion for the cantons) mainly reduces the consolidation requirement at the cantonal level. Nonetheless, measures that dampen expenditure growth in health care, such as the budgetary target proposed by the Federal Council, are still required to ensure sustainable financing (Brändle et al., 2018; Brändle and Colombier, 2020; Mattmann et al., 2021). The Confederation, on the other hand, has a relatively small need for consolidation.

Our analysis suggests that the COVID-19 crisis does not significantly affect fiscal sustainability. Even assuming that the COVID-19 crisis also has a long-term negative impact on economic performance and general government receipts, the 30-year debt ratio would rise to 51% of GDP instead of 45% in 2050. In other words, the state would have to consolidate an extra 0.3% of GDP a year to stabilise debt at the 2019 baseline year level.

Aside from the ageing of the population, climate change is an important long-term challenge for Switzerland. The effects of climate change are much more uncertain than the effects of ageing and therefore very hard to quantify. The qualitative analysis in this article discusses some channels through which climate change impacts public finances in terms of both receipts and expenditure. The overall effect of these impacts will be negative. Lower tax receipts and higher expenditure are to be expected, which will reduce fiscal room for manoeuvre. These considerations need to be explored in greater depth against the backdrop of advancing climate change.

To sum up, sustainable public finances are crucial for dealing with a crisis such as COVID-19 and the long-term challenges of population ageing and climate change. A benign macroeconomic environment such as steady productivity growth and low interest rates considerably lessens the burden on public finances.
